# Trajectories of Childhood Weight Gain: The Relative Importance of Local Environment versus Individual Social and Early Life Factors

**DOI:** 10.1371/journal.pone.0047065

**Published:** 2012-10-15

**Authors:** Megan A. Carter, Lise Dubois, Mark S. Tremblay, Monica Taljaard, Bobby L. Jones

**Affiliations:** 1 Institute of Population Health, University of Ottawa, Ottawa, Ontario, Canada; 2 Department of Epidemiology and Community Medicine, University of Ottawa, Ontario, Canada; 3 Healthy Active Living and Obesity Research, Children’s Hospital of Eastern Ontario Research Institute, Ottawa, Ontario, Canada; 4 Clinical Epidemiology Program, Ottawa Hospital Research Institute, Ottawa, Ontario, Canada; 5 Behavioral Genetics Research Program, Western Psychiatric Institute and Clinic, University of Pittsburgh Medical Center, Pittsburgh, Pennsylvania, United States of America; Pennington Biomedical Research Center, United States of America

## Abstract

**Objective:**

To determine the association between local environmental factors with child weight status in a longitudinal study, using a semi-parametric, group-based method, while also considering social and early life factors.

**Methods:**

Standardized, directly measured BMI from 4–10 y of age, and group-based trajectory modeling (PROC TRAJ) were used to estimate developmental trajectories of weight change in a Québec birth cohort (n = 1,566). Associations between the weight trajectories and living location, social cohesion, disorder, and material and social deprivation were estimated after controlling for social and early life factors.

**Results:**

Four weight trajectory groups were estimated: low-increasing (9.7%); low-medium, accelerating (36.2%); medium-high, increasing (43.0%); and high-stable (11.1%). In the low-increasing and medium-high trajectory groups, living in a semi-urban area was inversely related to weight, while living in a rural area was positively related to weight in the high-stable group. Disorder was inversely related to weight in the low-increasing group only. Other important risk factors for high-stable weight included obesity status of the mother, smoking during pregnancy, and overeating behaviors.

**Conclusions:**

In this study, associations between local environment factors and weight differed by trajectory group. Early life factors appear to play a more consistent role in weight status. Further work is needed to determine the influence of place on child weight.

## Introduction

The prevalence of childhood obesity has been increasing globally in the last few decades and is now a problem in both the developing and developed world [1]. In North America, childhood obesity has more than doubled from 1978/79 to 2004 among Canadian children aged 2–17 y [2] and almost tripled among American children aged 2–19 y in a similar time period [3]. Given this increasing prevalence and associations with adverse health outcomes, being at excess weight in childhood has become a major public health concern [4], with potentially far-reaching and long-lasting social and economic consequences [5].

Obesity research has typically focused on individual behaviours. While this is important, and may have played a role in the levelling off of childhood obesity in certain countries [6], it may not be enough to shift the entire population distribution of childhood obesity and related diseases downward. As such, there have been recent calls by the obesity research community to consider the environments in which people live [7]. This ecological, more holistic understanding of excess weight development will likely be necessary to develop interventions that are not only effective, but also sustainable at the population-level.

The level of material deprivation or disadvantage at the local level may explain whether healthy eating or physical activity resources will be available to residents, and at what cost [8]. This may have ramifications for child weight status. For example, studies assessing neighborhood-level material deprivation often find that children living in the most deprived areas are most likely to be overweight or obese [9]–[11]. The level of material deprivation may also confound the relationship between other place characteristics and childhood excess weight, and thus, is important to include in multivariable models.

Deprivation theorists also highlight the importance of including a social interaction aspect, emphasizing that deprivation cannot be reduced to a single material or economic dimension [12]. Social deprivation relates to the concepts of social capital, social fragmentation, and social isolation [12]. Areas with high social deprivation (fewer social ties) may decrease resident’s access to material and non-material resources conducive to healthy living, and increase stress and decrease well-being, all of which may contribute to child excess weight. To the authors’ knowledge, this has not yet been studied in relation to child excess weight.

Social capital has no agreed upon definition but has been extensively studied in relation to adverse health outcomes such as delayed child development [13]. One definition conceptualizes social capital as a group-level attribute called social cohesion. This refers to the resources – such as trust, norms, and the exercise of sanctions – available to the members of social groups [14].

Broadly speaking, posited ways that high social cohesion may prevent childhood excess weight include increased ability to link with health relevant resources, collective action to bring needed resources into the community, and the existence of social norms that enforce healthy behaviours and prevent deviant behaviors that may lead to degradation or disorder of the physical (e.g., garbage, graffiti) and social environments (e.g. fear, crime, bad reputation). The literature on social cohesion is small but growing. These studies, for the most part, have uncovered inverse associations with child excess weight [15]–[17].

Areas of increasing social disorganization, characterized by increasing disorder, may promote stress, discourage healthy behaviours among residents, such as being physically active outside, and itself may negatively impact social capital. It is sometimes considered a measure of neighbourhood quality. The few studies that have examined local area disorder in relation to childhood excess weight have found positive associations between disorder and child excess weight [10], [11], [18].

How developed an area is, with respect to living location or urban/rural status may also help to explain the development of childhood obesity. Rural areas have fewer amenities and they may be of lower quality than more populated areas. Additionally, as distance from the city centre increases, time spent in the car increases [19]. A representative cross-sectional study of Canadian children in grades 6–10 found that as the level of rurality increased, the odds of overweight and obesity increased [20]. Some studies have observed similar associations [21]–[23]. However, others have had opposite [24] or null results [25], [26].

Assessing potential local environmental relationships with child weight status is a relatively new and burgeoning area of research. The literature is heterogeneous and there is a dearth of studies that examine measures of social deprivation, social cohesion, and disorder. Regardless of the type of local environmental characteristic being examined, few studies have used the longitudinal research design with more than two repeated measures. This greatly limits what can be said about the childhood place – weight relationship. Previous work on child excess weight development has also highlighted the potential usefulness of statistical methods that do not assume all children follow the same growth trajectory, such as group-based trajectory modeling or growth mixture modeling [26]–[28]. These methods account for heterogeneity in growth by grouping children together based on similar response patterns over time. Partitioning variability in growth in this way may allow for a better understanding of how weight status changes over time and provides another means of detecting risk factors, which can complement the single growth curve approach, such as random effects modeling.

Therefore, the main objective of the present study was to use group-based trajectory modeling to examine change in living location and perceived neighbourhood social cohesion and disorder, in relation to change in child weight status, after controlling for area material and social deprivation and other potentially important explanatory variables. The main hypothesis was that adverse local environment factors such as rural living, high disorder, and high material and social deprivation would be related to increasing weight over time, and that high cohesion would be related to decreasing weight over time.

## Methods

### Sample

Children participating in the Québec Longitudinal Study of Child Development (QLSCD) comprised the sample for analysis. The QLSCD is a government-funded cohort study conducted by the Institut de la Statistique du Québec (ISQ) in the province of Québec, Canada. It began in 1998 with the intent of collecting data describing the health and well-being of Québec children from infancy into young adolescence [29]. Data collection began at five months of age and occurred annually until the age of eight, after which it occurred biannually, in order to reduce respondent burden. Cohort children are currently 14 years of age. Data from five months to 10 years of age were used in the current study.

Children were selected for participation in the QLSCD based on a three-stage, stratified design, using 1997–1998 Québec Birth Registries as the sampling frame [29]. Twins, children with major diseases at birth, and those living in Northern Québec, Cree or Inuit territory, or Native reserves were excluded. Sampling occurred throughout the year to minimize the effect of seasonality. Of the 2,675 children and their families that were possible to contact, 2,223 agreed to participate (83% response rate). A further 103 were dropped, as these were a cross-sectional over-sample for the first year (1998), to arrive at n = 2,120 for the longitudinal sample size. Computer-assisted personal interviewing of the mother, in the child’s home, was the primary method for data collection. The present study is secondary analysis of data collected in the QLSCD.

### Outcome Variable

The dependent variable used in the analyses was body mass index (BMI) Z-score. This is BMI (kg/m^2^) that is standardized, based on age and sex, to a reference population; in this case, the Centers for Disease Control and Prevention (CDC) 2000 Growth Charts [30]. Standardization of BMI is necessary since an increase in BMI is a part of normal growth. Heights and weights were directly measured at the approximate ages of 4, 6, 7, 8, and 10 years; details of how these measurements were taken can be found elsewhere [31].

### Local Environment Factors

Five variables describing the local environment were available for use in the QLSCD. The size of the local area described depends on particular variable definitions, and is not constant across variables. For instance living location covers large areas at the municipality level; neighbourhood social cohesion and disorder cover the mother’s perceived neighbourhood; and material and social deprivation cover census enumeration areas, which represent areas equivalent to one or more city blocks. These variables are described in more detail below, under three main subheadings. Raw area information such as postal codes was not available to use in the present analysis.

### Living Location

Children’s postal codes were linked to census geographical areas using Statistics Canada’s postal code conversion file by statisticians at the ISQ. For the purposes of this study, children were classified as living in one of three different types of areas: 1) Urban: Census Metropolitan Area (CMA) with ≥ 100,000 inhabitants; 2) Semi-urban: Census Agglomeration (CA) with 10,000 to <100,000 inhabitants; or 3) Rural: rural area/small town with <10,000 inhabitants [32]. This variable was considered time-dependent as it was measured at each data collection cycle. Postal codes were not collected at age 4 years, thus the living location value at the previous data collection cycle (3.5 years) was used at 4 years of age.

### Neighbourhood Social Cohesion and Disorder

Two scales (social cohesion, disorder), adapted from those previously used by Statistics Canada, were used in this analysis to characterize elements of neighbourhood physical and social environments [29], [32]. Social cohesion measures the level of mutual trust and support that neighbours have for one another. The mother was asked to give her level of agreement to five statements, which were then used to derive an average score ranging from one to four; lower scores indicate higher cohesion. Disorder measures the overall quality of the neighbourhood. The mother was asked to assess the presence and severity of four types of problems in the neighbourhood. Responses were then used to derive an average score ranging from one to three; a score of three indicates no problems. Both scales have demonstrated adequate internal consistency, as each was originally calculated to have a Cronbach’s alpha of ≥0.75 [33]. Items for each scale can be found in Appendix S1. Both scales were dichotomized to increase interpretability. In order to maximize efficiency and minimize bias, this was based on the 50^th^ percentile. However, disorder was highly skewed and instead, children were categorized as either having a perfect score of three (no problems at all in the neighbourhood) versus less than three (problems present). A similar approach was taken by Curtis et al (2004) in their analysis of neighbourhood influences on a variety of health outcomes in a Canadian sample of children [34].

These variables were collected every other data collection cycle and thus available at 4, 6, 8, and 10 years of age. To be considered a time-dependent variable in analysis, a value at 7 years was needed; thus, the value at 6 years was carried forward.

### Deprivation

Material and social deprivation are two separate dimensions of a deprivation index developed to aid the Québec Government in assessing community service needs and monitoring social inequalities [35], [36]. Material deprivation measures the inability to get the goods and services that are a part of everyday life (lack of human and economic capital), and social deprivation captures social isolation within the neighbourhood. This index has been derived for QLSCD children and was available for secondary data analysis. Derivation of the index in the QLSCD is discussed below.

Children’s postal codes, measured in 1998 (first data collection cycle when children were five months of age), were linked to 1996 census data for residents ≥15 years of age aggregated to the enumeration area level. The enumeration area was chosen because it was the smallest geographical unit for which census data were available in the 1996 Canadian census (with an average population of 750 residents) [36].

Principal components analysis was then conducted by ISQ statisticians to determine factor scores for each of the two dimensions, based on the aggregated census data at the enumeration level. Material deprivation factor scores were calculated from mean income, percentage of residents with no high school diploma, and ratio of employed residents to total population. Social deprivation factor scores were calculated from percentage of single-parent families, percentage of families split by separation, divorce, or death, and percentage of residents living alone. On both dimensions, enumeration areas were divided into population quintiles, from quintile 1 (least disadvantaged) to 5 (most disadvantaged).

For the present secondary data analysis, both material and social deprivation were dichotomized into ‘deprived’ (quintiles 4 and 5) versus ‘not deprived’ (quintiles 1–3), as has been done elsewhere [37] (Table 1). Both were also considered time-stable in analyses as they were not available in other cycles, mainly because the Canadian census is conducted once every five years. An update, based on the 2001 census, linked to postal codes was not available for this study, but descriptive data showed that these measures remained largely unchanged from 1996–2001 [38]. Children could also move to a different enumeration area (this became dissemination area in the 2001 census) over time; however, these changes in material deprivation and social deprivation were not derived in the QLSCD every time a child moved.

**Table 1:** Definitions of explanatory variables.

Variable	Definition	Timing of data collection(age of child)^a^
*Individual-level*		
Male sex	Yes/no	5 months
Overeating phenotype	‘Often’ eats too much and/or ‘sometimes’ or ‘often’ eats too fast	4 years
Breast-fed exclusively ≥ 3 months	Yes/no	5 months
Mother smoked during pregnancy	Yes/no	5 months
Mother’s obesity status (WHO)	BMI ≥30 based on self-reported height and weight	1.5 years
High birth weight	>4 kg vs ≤ 4 kg	5 months
Rapid weight gain 0–5 months	Highest two quintiles of average monthly weight gain from 0 to 5 months	5 months
*Family/household*		
Low socioeconomic status (SES)b c	Lowest third of SES indicator versus middle and upper tertiles	Time-dependent
Single parent family	Yes/no	Time-dependent
Mother is an immigrant	Yes/no	5 months
*Local environment*		
Living location^b^	Categorical (Urban: Census Metropolitan Area with ≥ 100,000 inhabitants; Semi-urban: Census Agglomerations with 10,000 to <100,000 inhabitants; and Rural: Rural or Small Towns with <10,000 inhabitants)	Time-dependent
High social cohesion^d^	Scale score in the bottom 50%	Time-dependent
High disorder^d^	Scale score <3	Time-dependent
Materially deprived	Two highest factorial score quintiles (4 and 5)	5 months
Socially deprived	Two highest factorial score quintiles (4 and 5)	5 months

a Time-dependent indicates that these variables were available at 4, 6, 7, 8, and 10 years of age and thus were treated as time-dependent explanatory variables in the analysis. If a variable was only measured once and occurred at or before baseline (4 y) it was treated as a ‘risk factor’ (time-stable).

b Missing at 4 y of age for all children, value at age 3.5 y was carried forward to age 4.

c For more information on how this variable was calculated and interpreted, please see reference 32.

d Measured every other data collection cycle for all children (value at age 6 was carried forward for age 7).

### Statistical Analysis

Group-based trajectory modeling (sometimes called latent class growth analysis or semi-parametric finite mixture modeling) [39] was employed to determine relationships between the various local environment factors and weight status, using PROC TRAJ in SAS (v. 9.2) [40], [41]. Group-based trajectory modeling assumes that there are a certain number of discrete underlying groups in the population that each have their own population prevalence, intercept and slope (trajectory shape or change in BMI Z-score) [39]. These subpopulations are not directly observable, but are estimated (latent) and should not be considered actual categories of growth. Rather, they are used to help us understand the etiological underpinnings of different developmental trajectories [42].

In general terms, the series of responses for each individual are used to determine subgroups that follow similar changes in the outcome over time. This is done in a probabilistic manner, using maximum likelihood [39]. The linkage between age (time) and the outcome occurs through a polynomial relationship that is estimated via a latent variable. Additionally, the probability of belonging to each group is calculated for each individual and is estimated from the model parameters. These are the posterior probabilities and they give an overall estimate of model uncertainty. For descriptive analyses and model-checking purposes, individuals can be assigned to the group to which they have the highest probability of belonging.

The first step involved determining the most appropriate number of groups and the shape of their trajectories. This was done by comparing Bayesian Information Criteria (BIC) for models with one to five groups; the more complex model (one with more groups) was considered a better fit than the null model (one with fewer groups) when two times the change in the BIC was equal to or larger than previously established criteria [40], [43]. After selection of the number of groups, the trajectory shapes of each of the groups was determined in a step-wise manner starting with all groups set to have a cubic order, and again comparing change in BIC and the significance of parameters, as their orders were made less complex – quadratic, linear and intercept only. Sometimes the BIC is not a good indicator of fit and will continue to decrease as more and more groups are added, even though the additional groups do not add any extra explanatory power. Therefore, the best fitting models for three, four, and five groups were compared graphically. In order to test for the presence of interactions, these steps were also carried out separately by sex to determine if the same number of groups with similar trajectory shapes emerged. The resulting final model was then assessed for goodness of fit based on standard criteria, namely: the average posterior probabilities for each of the subgroups, odds of correct classification, and comparison of actual (based on posterior probabilities) to model/estimated group prevalences [43]. According to Nagin (2005), rules of thumb for assessing adequate fit are that each group should have an average posterior probability ≥0.7 and an odds of correct classification ≥5, and there should be close agreement between actual and estimated group prevalences [43].

Next, profiles of each of the trajectory groups were created to explore differences in the local environment factors, other explanatory factors, and outcome at a descriptive level (means and proportions). No formal statistical tests were conducted at this point, as the uncertainty of group assignment needs to be taken into account by using the posterior probabilities [43].

The last step of modelling was to relate the local environment factors to the trajectories while controlling for social and early life factors. Table 1 describes the treatment of all variables in model estimation. The time-dependent explanatory variable parameters are interpreted the same way they would be in an ordinary linear regression, but within each trajectory group. In other words, time-dependent explanatory variables may be associated with a deviation in the long-term average response (BMI Z-score) of members in a particular group [43]. Regressors can be different at different time points depending on changes in the time-dependent explanatory variable. Time-stable explanatory variables (risk factors) are related to the trajectory groups via a generalized logit function [40]. Resulting log-odds can be exponentiated to get odds ratios, which are interpreted as odds of being in a certain trajectory group relative to the reference group for a level of a risk factor versus the reference level.

Unadjusted models were first estimated for the local environment factors separately, then a model with local environment and social variables together, and a final model that added early-life variables. All analyses take into consideration the uncertainty of group assignment, and in multivariable models, estimate parameters for time-dependent and time-stable explanatory variables simultaneously.

Group-based trajectory modeling retains subjects with partially observed outcome and time-dependent explanatory variable data under the assumption that data are missing at random [42]. Subjects with any missing values on one or more time-stable explanatory variables, or those who are missing all data for a time-dependent explanatory variable are dropped from the analysis automatically. For this particular analysis, participants that had fewer than two out of the five BMI Z-score outcome variables non-missing were excluded.

Due to the cumulative effect of missing data for the explanatory variables, 1385 children were included in the multivariable model that included all explanatory variables (local environment, and social and early life factors). Compared to included children, excluded children (n = 735) were more likely to be male, have immigrant mothers, be from a low SES household, and experience rapid weight gain during infancy (χ^2^ *P*-values <0.05). Of the excluded children that had BMI Z-score measures, no significant differences with included children were noted at any of the time points.

Sampling weights were not used due to the fact that children could be included even if they did not respond in all cycles. Therefore, the results here cannot be considered representative of the Québec population.

### Ethics Statement

Each year, participants were provided with detailed information on the aims and procedures of the QLSCD, and consent was obtained by the interviewer from either one or both parents of the child, using a form approved by the Ethics Committee of the ISQ. Ethics approval to conduct this secondary analysis was given by the University of Ottawa Research Ethics Board - certificate number: H 05-10-18.

## Results

### Descriptive Trajectory Data

Approximately 74% of children (n = 1566/2120) had two response points and were included in the first step of analysis: estimation of the number of groups and the shapes of their trajectories. According to the BIC tests, the 5-group model best fit the data, followed by the 4-group model. When fitting the trajectory shapes for the 5-group model, the prevalence of the fifth group was approximately 2%, which reduces interpretability and power. This model was graphically compared to the best fitting 3- and 4-group models. The parameter estimates of the 5-group model appeared to collapse onto those of the fourth group, so for parsimony, the 4-group model was selected as the best fitting model. Model fit statistics indicated an adequately fitting model (Appendix S2– group labels were assigned subjectively based on their trajectory shapes).

**Figure 1:**
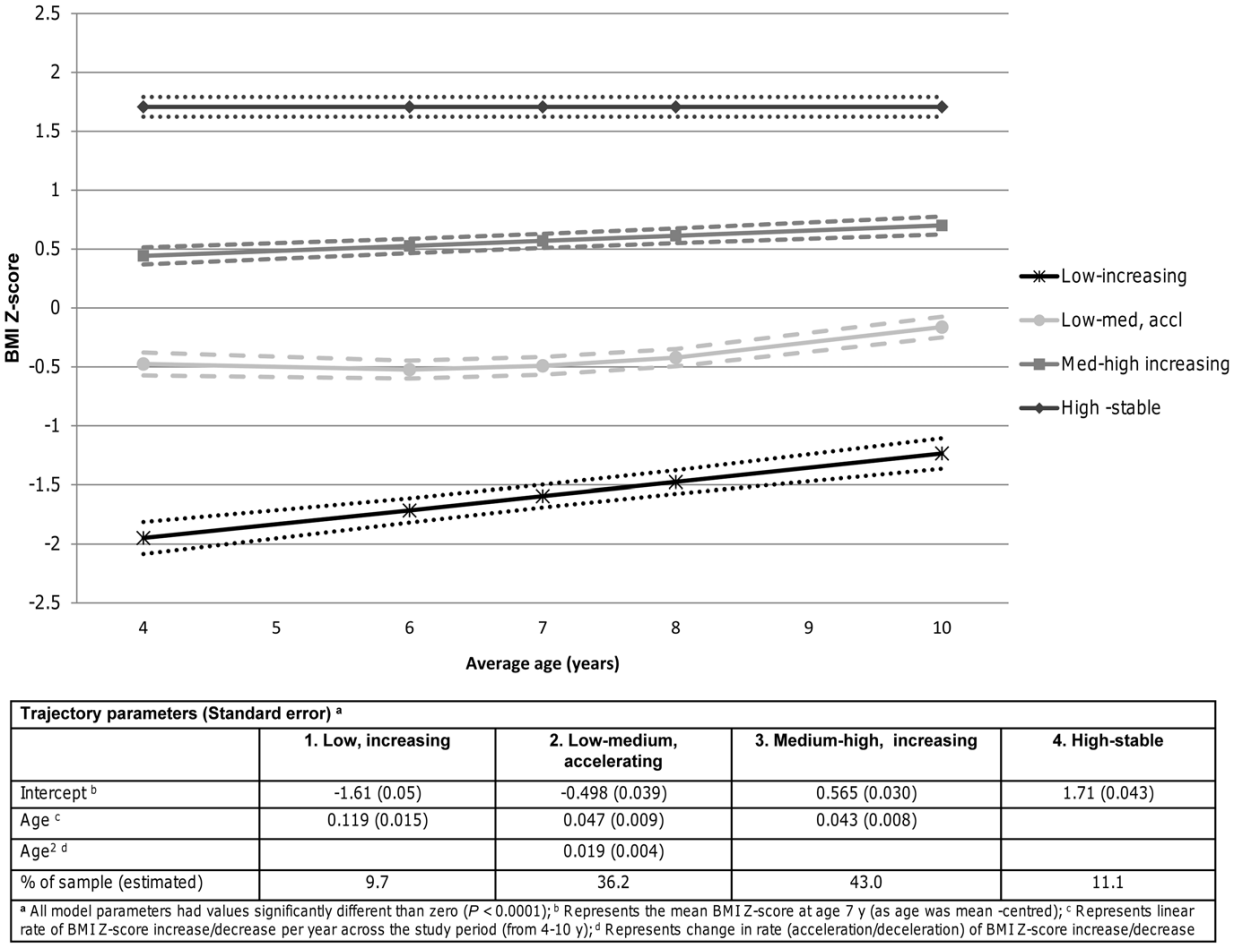
Estimated weight change trajectories and 95% confidence intervals in the QLSCD from 4–10 y of age (n = 1566).

Table 2 and Figure 1 present descriptive information for the four trajectory groups. On average, children in the low group were underweight at baseline, with a Z-score < −1.645. Growth was best explained by a positive linear relationship with time. This group was therefore labelled “low, increasing.” The second group, labelled “low-medium, accelerating” had a mean BMI Z-score below the median at baseline. Trajectory of growth was estimated to be quadratic over time. Group 3, labelled “medium-high, increasing” began slightly above the median at baseline and were estimated to have a linear trajectory. Finally, an intercept only relationship with time was estimated for the fourth group, labelled “high-stable,” indicating that their weight did not change significantly from ages 4–10 years (flat line trajectory). Mean BMI Z-score for these children at baseline was above 1.645 Z-score units (95^th^ percentile), which classifies them as obese according to the CDC definition [30], [44]. The majority of children were estimated to belong to the low-medium or the medium-high groups. When categorizing each group as overweight (≥ 85^th^ percentile) or obese (≥ 95^th^ percentile) at each age, no children in the low group and ≤ 3% of children in the low-medium group were overweight or obese at any age. In the medium-high group, percentage overweight ranged from 16–33% and percentage obese from 2–7% across the five ages. Finally, in the high-stable group, percentage overweight ranged from 85–99%, and percentage obese from 53–67% across the five ages.

**Table 2:** Mean BMI and BMI Z-score by age and weight trajectory group (N = 1566).

	Groups								
	1. Low, increasing		2. Low-medium, accelerating		3. Medium-high, increasing		4. High-stable		Total N
Variable	Mean (SD)	N	Mean (SD)	N	Mean (SD)	N	Mean (SD)	N	
**BMI Z-score 4 y**	−1.90 (0.88)	118	−0.50 (0.78)	489	0.53 (0.79)	581	1.73 (0.71)	134	1322
**BMI 4 y**	13.7 (0.71)		15.0 (0.84)		16.2 (1.11)		18.4 (1.85)		
**BMI Z-score 6 y**	−1.86 (1.00)	102	−0.55 (0.72)	407	0.52 (0.60)	497	1.73 (0.55)	116	1122
**BMI 6 y**	13.5 (0.84)		14.7 (0.87)		16.2 (1.06)		19.4 (2.20)		
**BMI Z-score 7 y**	−1.67 (0.93)	133	−0.57 (0.68)	540	0.52 (0.56)	627	1.73 (0.52)	149	1449
**BMI 7 y**	13.7 (0.83)		14.8 (0.88)		16.5 (1.14)		20.5 (2.86)		
**BMI Z-score 8 y**	−1.65 (0.91)	126	−0.40 (0.64)	477	0.66 (0.54)	566	1.83 (0.39)	135	1304
**BMI 8 y**	13.8 (0.87)		15.3 (0.99)		17.4 (1.40)		21.9 (2.65)		
**BMI Z-score 10 y**	−1.15 (0.64)	116	−0.18 (0.63)	471	0.77 (0.61)	532	1.77 (0.46)	134	1253
**BMI 10 y**	14.9 (0.95)		16.6 (1.41)		19.2 (2.2)		24.3 (3.3)		

Conducting a stratified analysis by sex showed no differences in the number of groups or trajectory shapes compared to the original aggregate estimate. This indicates that there were likely no substantial differences (interactions) in development by sex. Thus, to increase power, males and females were analyzed together. Descriptive information describing the characteristics of the trajectory groups are presented in Table 3.

**Table 3:** Characteristics of QLSCD children by weight trajectory group (Total N = 1566).

	Groups					
	1. Low, increasing	2. Low-medium, accelerating	3. Medium-high, increasing	4. High-stable	Total	Missing N
*Social factors*						
Male	54.6 (78)	46.9 (272)	46.9 (319)	50.3 (82)	48.0	0
SES^b^						
Low	28.5 (39)	31.2 (177)	31.0 (208)	46.9 (75)	32.5	30
Middle	35.8 (49)	34.3 (195)	34.3 (230)	28.8 (46)	33.9	
High	35.8 (49)	34.5 (196)	34.7 (233)	24.4 (39)	33.7	
Single parent family^b^	12.1 (17)	12.9 (73)	13.0 (87)	16.2 (26)	13.2	27
Mother is an immigrant	7.7 (11)	7.2 (42)	9.3 (63)	9.2 (15)	8.4	1
*Selected early life factors*						
Child overeats	17.0 (24)	16.0 (91)	22.3 (150)	54.7 (88)	22.9	23
Mother smoked during pregnancy	26.2 (37)	24.9 (144)	23.2 (157)	36.4 (59)	25.5	8
Exclusively breast-fed ≥ 3 months	24.5 (35)	25.0 (145)	27.4 (186)	23.9 (39)	25.9	0
Rapid weight gain in infancy	31.9 (45)	34.6 (197)	41.1 (274)	46.5 (74)	38.4	31
*Local environment*						
Living location^b^						
Rural	25.6 (35)	25.3 (143)	19.8 (132)	26.1 (42)	23.0	34
Semi-urban	6.6 (9)	12.9 (73)	13.2 (88)	10.6 (17)	12.2	
Urban	67.9 (93)	61.8 (350)	67.1 (448)	63.4 (102)	64.8	
High social cohesion^b^	50.4 (70)	49.7 (274)	45.8 (293)	49.4 (76)	48.1	82
High disorder^b^	24.1 (34)	25.0 (141)	27.5 (184)	23.8 (38)	25.9	32
Materially deprived	31.9 (43)	38.4 (209)	38.3 (245)	43.5 (67)	38.3	93
Socially deprived	31.1 (42)	34.7 (189)	39.3 (251)	42.2 (65)	37.1	93

a Values are percentages (n).

b Values presented are those measured at baseline (when children were approximately 4 y of age).

### Relationship between Local Environment Factors and Weight Trajectories

In bivariable and multivariable analyses, none of the time-stable local environment factors (material and social deprivation) were associated with group membership (Table 4). On the other hand, overeating, having a mother that smoked during pregnancy, or was obese when the child was young, greatly increased the odds of following the high-stable trajectory relative to the ‘normal’ (medium-high, increasing) group. Rapid weight gain, high birth weight, and having an obese mother decreased the odds of children belonging to the two lower trajectory groups, relative to the normal group.

**Table 4:** Results of unadjusted and multivariable group-based trajectory models estimating the relationship between time-stable explanatory variables and probability of group membership, and change in average group BMI Z-score as a function of time-dependent explanatory variables (QLSCD children from 4–10 y of age).

	Unadjusted	Model 1 (n = 1472)^a^	Model 2 (n = 1385)^b^
**Time-stable explanatory variables: Odds ratios (95% CI)** c			
*Local environment*High Material Deprivation			
1. Low, increasing	0.79 (0.52, 1.21)	0.66 (0.41, 1.06)	0.64 (0.39,1.06)
2. Low-med, accelerating	0.95 (0.71, 1.26)	0.91 (0.66, 1.24)	0.92 (0.67, 1.28)
3. Med-high, slow increasing – Ref	–	–	–
4. High-stable	1.30 (0.86, 1.96)	1.09 (0.71, 1.67)	0.91 (0.57, 1.45)
High Social Deprivation	–	–	–
1. Low, increasing	0.70 (0.46, 1.07)	0.75 (0.47, 1.18)	0.69 (0.43, 1.12)
2. Low-med, accelerating	0.78 (0.58, 1.04)	0.75 (0.55, 1.02)	0.72 (0.53, 0.99)*
3. Med-high, slow increasing – Ref	–	–	–
4. High-stable	1.07 (0.71, 1.62)	1.03 (0.67, 1.57)	0.91 (0.57, 1.44)
*Social factors*			
Male sex			
1. Low, increasing	1.32 (0.89, 1.94)	1.30 (0.86, 1.97)	1.62 (1.04, 2.53)*
2. Low-med, accelerating	0.97 (0.74, 1.28)	1.01 (0.75, 1.36)	1.24 (0.91, 1.68)
3. Med-high, slow increasing - Ref	–	–	–
4. High-stable	1.14 (0.77, 1.69)	1.14 (0.76, 1.73)	1.14 (0.72, 1.80)
Mother is an immigrant			
1. Low, increasing	0.87 (0.44, 1.73)	0.87 (0.39, 1.92)	0.53 (0.20, 1.42)
2. Low-med, accelerating	0.63 (0.36, 1.07)	0.82 (0.46, 1.46)	0.82 (0.45, 1.48)
3. Med-high, slow increasing – Ref	–	–	–
4. High-stable	1.12 (0.58, 2.15)	1.04 (0.48, 2.25)	0.81 (0.31, 2.07)
*Early life factors*			
Overeating phenotype			
1. Low, increasing	0.74 (0.44, 1.23)		0.94 (0.53, 1.68)
2. Low-med, accelerating	0.63 (0.44, 0.90)*		0.76 (0.51, 1.14)
3. Med-high, slow increasing - Ref	–		–
4. High-stable	5.07 (3.32, 7.75)***		5.09 (3.18, 8.15)***
Mother smoked during pregnancy			
1. Low, increasing	1.44 (0.92, 2.24)		1.39 (0.83, 2.34)
2. Low-med, accelerating	1.16 (0.84, 1.61)		1.05 (0.73, 1.53)
3. Med-high, slow increasing – Ref	–		–
4. High-stable	2.31 (1.52, 3.50)***		2.51 (1.53, 4.12)**
Mother is obese			
1. Low, increasing	0.18 (0.05, 0.62)**		0.26 (0.09, 0.79)*
2. Low-med, accelerating	0.41 (0.24, 0.70)**		0.28 (0.15, 0.53)***
3. Med-high, slow increasing - Ref	–		–
4. High-stable	2.25 (1.39, 3.63)**		2.45 (1.39, 4.29)**
Breastfed exclusively (> = 3 months)			
1. Low, increasing	0.77 (0.49, 1.22)		0.79 (0.47, 1.32)
2. Low-med, accelerating	0.82 (0.61, 1.11)		0.82 (0.58, 1.15)
3. Med-high, slow increasing - Ref	–		–
4. High-stable	0.75 (0.48, 1.19)		1.12 (0.66, 1.91)
Rapid weight gain during infancy			
1. Low, increasing	0.70 (0.46, 1.05)		0.46 (0.29, 0.75)**
2. Low-med, accelerating	0.73 (0.55, 0.97)*		0.63 (0.45, 0.87)**
3. Med-high, slow increasing – Ref	–		–
4. High-stable	1.27 (0.85, 1.88)		1.35 (0.83, 2.19)
High birth weight (>4 kg)			
1. Low, increasing	0.22 (0.08, 0.63)**		0.21 (0.07, 0.63)**
2. Low-med, accelerating	0.52 (0.33, 0.81)**		0.49 (0.29, 0.81)**
3. Med-high, slow increasing - Ref	–		–
4. High-stable	1.04 (0.59, 1.86)		1.07 (0.53, 2.15)
**Time-dependent explanatory variables: Linear regression parameter (SE)** d			
*Local environment*			
Living location (Rural)^e^			
1. Low, increasing	−0.149 (0.093)	−0.187 (0.123)	−0.215 (0.120)
2. Low-med, accelerating	0.023 (0.058)	0.026 (0.083)	−0.028 (0.073)
3. Med-high, slow increasing	0.079 (0.060)	0.054 (0.072)	−0.011 (0.059)
4. High-stable	0.274 (0.086)**	0.257 (0.097)**	0.208 (0.089)*
Living location (Semi-urban)^e^			
1. Low, increasing	−0.421 (0.158)**	−0.453 (0.154)**	−0.541 (0.161)**
2. Low-med, accelerating	0.0480 (0.065)	0.053 (0.067)	0.0415 (0.068)
3. Med-high, slow increasing	−0.148 (0.057)**	−0.111 (0.063)	−0.134 (0.059)*
4. High-stable	0.034 (0.098)	0.045 (0.111)	−0.008 (0.107)
High social cohesion			
1. Low, increasing	0.114 (0.07)	0.160 (0.076)*	0.126 (0.082)
2. Low-med, accelerating	0.020 (0.037)	−0.015 (0.039)	−0.005 (0.040)
3. Med-high, slow increasing	−0.020 (0.033)	−0.021 (0.036)	−0.015 (0.036)
4. High-stable	−0.100 (0.062)	−0.064 (0.066)	−0.046 (0.067)
High disorder			
1. Low, increasing	−0.171 (0.081)*	−0.175 (0.086)*	−0.176 (0.088)*
2. Low-med, accelerating	−0.073 (0.041)	−0.060 (0.043)	−0.071 (0.044)
3. Med-high, slow increasing	0.017(0.036)	0.015 (0.040)	0.025 (0.040)
4. High-stable	0.045 (0.07)	0.027 (0.074)	0.0108 (0.075)
*Social Factors*			
Single-parent family			
1. Low, increasing	−0.046 (0.098)	0.051 (0.106)	0.009 (0.114)
2. Low-med, accelerating	−0.028 (0.054)	0.039 (0.061)	0.017 (0.062)
3. Med-high, slow increasing	0.137 (0.048)**	0.133 (0.053)*	0.094 (0.055)
4. High-stable	0.177 (0.088)*	0.138 (0.107)	0.116 (0.100)
Low SES			
1. Low, increasing	−0.207 (0.079)**	−0.129 (0.094)	−0.179 (0.093)
2. Low-med, accelerating	−0.102 (0.045)*	−0.109 (0.051)*	−0.106 (0.052)*
3. Med-high, slow increasing	0.007 (0.039)	−0.025 (0.045)	−0.064 (0.043)
4. High-stable	0.138 (0.066)*	0.055 (0.079)	−0.001 (0.079)

a Place factors plus social factors, model includes both time-stable (risk factors) and time-dependent explanatory variables.

b Model 1, plus adjustment for early life factors; model includes both time-stable (risk factors) and time-dependent explanatory variables.

c Odds ratios and 95% confidence intervals from multinomial logistic regression, reference group is the medium-high, slow increasing group.

d Parameters represent the average increase/decrease in BMI Z-score per year within each trajectory group based on a polynomial link function.

e Reference group is Urban.

* *P≤* 0.05, **P ≤ 0.01 *** *P ≤* 0.0001; CI  =  confidence interval; SE  =  standard error.

Among the time-dependent local environment factors, living in a rural area relative to an urban area (CMA) was associated with an increase in BMI Z-score among children in the high-stable group (0.274, 95%CI: 0.105 to 0.443) (Table 4). This attenuated slightly in the multivariable analyses.

Living in a semi-urban area (CA) relative to an urban area (CMA) was associated with a decrease in BMI Z-score of the low and medium-high groups by 0.421 (95%CI: −0.111 to −0.730) and 0.148 (95%CI: −0.260 to −0.036) units, respectively. Multivariable analyses slightly attenuated the association among the medium-high group, and strengthened the association in the low group.

High social cohesion was not significantly associated with change in weight in any group. High disorder was significantly associated with change in weight in the low group only. Living in this type of environment was associated with a mean decrease of 0.171 (95%CI: −0.330 to −0.012) in BMI Z-score relative to not living a high disorder area. Multivariable analyses slightly strengthened this association.

**Figure 2:**
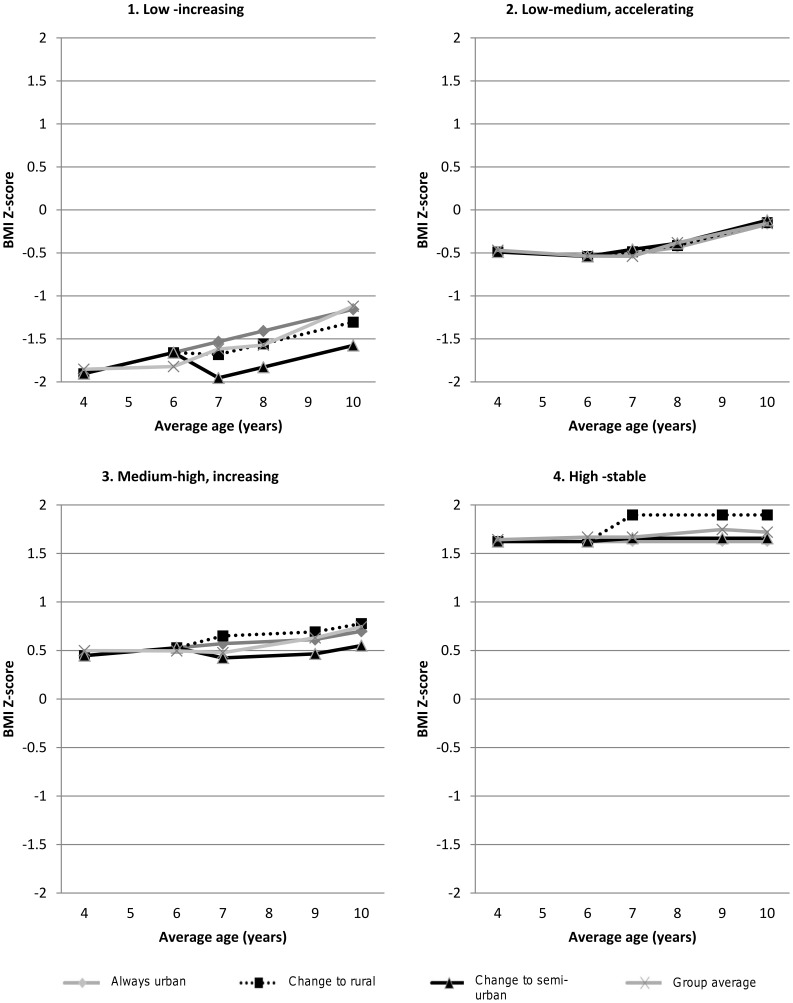
Example of time-varying local environment associations: effect of change in living location between 6–7 y of age on trajectory group shape (unadjusted).

Of the other time-dependent explanatory variables, low SES was related to decreasing weight in the low-medium accelerating group only. Figure 2 provides an example, using parameter estimates, of how moving from an urban (CMA) to a rural area or from an urban (CMA) to a semi-urban (CA) area between 6–7 years relates to changes in BMI Z-score in each of the four groups.

## Discussion

Four distinct trajectories of relative weight development were estimated in this cohort of children. A recent review of similar studies using group-based trajectory modeling or growth mixture modeling determined that four population subgroups were most common, with stable high and low categories, and rising and declining categories [45]. Most of the reviewed studies estimated trajectory groups based on a dichotomous indicator, such as overweight or obesity, or BMI without adjustment for age and sex. Dichotomizing the outcome can create misclassification bias in its own right, and adds to the classification uncertainty inherent in this type of analysis. Using BMI, without adjustment for age and sex, may risk missing, and therefore not accounting for, important sources of variation in growth. BMI Z-scores and percentiles, resulting from standardization to a reference distribution, are interchangeable [46]; however, in longitudinal population-based analyses BMI Z-scores have been preferred over use of percentiles [47].

In contrast to the results here, a recent study that examined the same cohort from 5 months to 8 years of age, uncovered three rather than four groups [48]. Approximately 5% of children were estimated to be in a high-rising BMI group, 41% in the moderate BMI group, and over half of the sample was estimated to belong to the low-stable group. This study, conversely, used both mother-reported and directly measured heights and weights. A previous study on this population reported inaccuracies in mother-reported height and weight [49]. This, along with using BMI that was not standardized for age and sex, and analyzing a different time-frame (5 months –8 years of age versus 4–10 years of age) may explain differences with the present study.

Eleven percent of children in the present study were estimated to follow a high-stable weight trajectory (group 4). This suggests that in some children, excess weight starts early, with certain risk factors that occur at or before 4 years of age, greatly increasing the likelihood of a child following this pattern of growth. In particular, this study highlights obesity status of the mother and smoking during pregnancy as potential points of intervention.

Among the other trajectory groups in the present study, there were no steep increases in weight, although all showed gradual increases in BMI Z-score over time. Early life factors related to obesity, such as high birth weight, rapid weight gain during pregnancy, and obesity status of the mother were less likely in the low and low-medium versus the medium-high weight change group.

Other than rural living in the high-stable group, local environment factors were either not related to weight or in an opposite direction of what was expected. However, family SES was also controlled for over time, so any influence of family SES on weight that coincided with moving should be controlled for. It is unclear why rural living would increase weight in the high-stable group and not the others. Perhaps children already at excess weight are more sensitive to changes in urban/rural lifestyle, and therefore exhibit weight changes more quickly [50]. Living in a semi-urban area (CA) was related to decreasing weight in the low and medium-high weight groups. This is also unclear and might indicate influences that go beyond population size. For example, the semi-urban areas considered here (CA) have fewer inhabitants than urban (CMA) but they have an urban core [51]. Thus, they may function as the best of both worlds, providing a sense of community due to their smaller size, but also a range of services.

Longitudinal studies on living location and child weight status have been mixed [21], [26]. A recent study that used growth mixture modeling to estimate relative weight change trajectories in a large US cohort of adolescents (12–17 years in 1997) did not find that living location (central metropolitan statistical area vs non-central metropolitan) predicted group membership [26]. The authors, however, did not account for change in this variable over time, used self-reported height and weight, and did not standardize BMI for age and sex. A longitudinal study of young Canadians that used a single growth curve approach, found that urban living was inversely related to weight status at initial status only; no differences in trends over time were noted [21]. Again, changes in living location could not be accounted for in this study.

Few studies in children have specifically examined the concept of disorder in relation to weight or obesity. In general, these studies have uncovered positive associations; increasing disorder being significantly related to increasing weight and/or an increased likelihood of obesity [10], [11], [18]. This is in contrast to the results of this study where no positive associations were seen; only an inverse association in the low weight group. On the other hand, two of these studies were cross-sectional [11], [18]. The third study was longitudinal and used a single growth curve method; although, significant results were for the cross-sectional association only, and the study was unable to adjust for changes in disorder over time [10]. Additionally, all of these studies were conducted in the US. Two of these studies included aspects of physical and social disorder (e.g. dilapidated buildings and teens hanging out) [10], [11] and one appeared to focus on physical disorder only [18]. The present study includes measures of both, although three out of the four items relate more to the social aspect of disorder, while only one captures the physical aspect of disorder. Two of the three studies were also interviewer observed [11], [18], rather than perceived by the respondent, as was the case in the present study.

Neighbourhood material deprivation was not related to weight status in this study. This is in contrast to fairly consistent findings from both cross-sectional and longitudinal studies that have shown positive relationships between area material disadvantage and childhood weight [9]–[11]. Social deprivation was also not found to relate to weight status in this study. No other studies, to the knowledge of the authors, have assessed this variable in relation to child weight status. Another study that examined area social deprivation in relation to overweight among adults living in Québec, did not uncover a significant association [37].

Social cohesion was also not related to weight status in this study. Previous studies on neighbourhood social cohesion that have uncovered inverse associations have been, for the most part, cross-sectional. A recent longitudinal study of Australian children found that neighbourhood parent-perceived social cohesion was inversely related to children’s BMI [17]. This study was similar to the present study in that social cohesion was a perceived, not an objective measure, and the items included in the measure of social cohesion are closely related to those studied here. On the other hand, this study did not adjust for household or neighbourhood SES, or standardize BMI for age and sex. Another study conducted in Los Angeles (US) found that collective efficacy (measures both social cohesion and informal social control) was inversely related to excess weight (BMI, overweight, obesity) [16]. When comparing to the null results here, it is important to note that this study could not separate the effects of social cohesion and informal social control.

The results of the present study should be interpreted in light of some important limitations. Due to the study design, changes in area material and social deprivation could not be taken into account. Due to missing data, the final model was estimated using only 65% of the original cohort; therefore, bias cannot be ruled out as an explanation for findings here. Additionally, because the analysis did not use sample weights, the results are not necessarily generalizable to the Québec population. Mediation and moderation were also not explored. Moderation (interaction) is important to more adequately explain complexity, as the relationship between the local environment and child weight might depend on other contextual and compositional factors. Mediation is important to more clearly explain and test hypothesized causal pathways. Unfortunately, both add a large degree of modeling complexity; especially problematic in a longitudinal study. Finally, differences between the results seen here and those of other studies may be due to the fact that the local environment was not adequately captured via the variables available in the QLSCD.

Although there were some limitations to this study, there were also numerous strengths. The longitudinal, population-based design, spanning a large portion of early childhood, was a major strength. A plethora of developmental data was available in this cohort and allowed us to adjust for a number of important explanatory variables. Height and weight were directly measured by trained interviewers; mother-reported child anthropometric measures have been found to overestimate overweight and obesity, especially at younger ages [52].

The results here show that early life factors may be most important for explaining excess weight among young children. Longitudinal studies on local environment factors may not corroborate the findings of earlier cross-sectional studies, as may be the case here. And relationships may be different at certain life periods, perhaps due to exposure time. More high quality, longitudinal studies on the local environment and childhood obesity development are warranted; especially those that can examine changes from birth to late childhood/early adulthood. Examining the mother’s local environment, in addition to behavioural factors, during the perinatal period may also be a fruitful area of obesity research.

### Disclaimer

This analysis was based on the Institut de la Statistique du Québec QLSCD master files. All computations were prepared by MAC. The responsibility for the use and interpretation of these data is solely that of the authors. The opinions expressed in this paper are those of the authors and do not represent the views of the Institut de la Statistique du Québec.

## Supporting Information

Appendix S1 **Neighbourhood Social Scale Items.**
(DOC)

Appendix S2 **Fit statistics for the trajectory groups estimated in the QLSCD.**
(DOCX)
