# Supplementary material for: Trajectories of Childhood Weight Gain: The Relative Importance of Local Environment versus Individual Social and Early Life Factors
Source: PLoS One. 2012 Oct 15;7(10):e47065. doi: 10.1371/journal.pone.0047065 (PMC3471956; doi:10.1371/journal.pone.0047065)
Supplement: Appendix S1 — Neighbourhood Social Scale Items. (DOC) [file pone.0047065.s001.doc]

| **Appendix A. Neighbourhood Social Scale Items** |  |
| --- | --- |
| **Scale** | **Response categories** |
| *Social Cohesion* |  |
| Please tell me whether you strongly agree, agree, disagree, or strongly disagree about the following statements… |  |
| 1. If there is a problem around here, the neighbours get together to deal with it | Strongly agree = 1 |
| 1. There are adults in the neighbourhood that children can look up to | Agree = 2 |
| 1. People around here are willing to help their neighbours | Disagree = 3 |
| 1. You can count on adults in this neighbourhood to watch out that children are safe and don't get in trouble | Strongly disagree = 4 |
| 1. When I'm away from home, I know that my neighbours will keep their eyes open for possible trouble |  |
| *Disorder* |  |
| How much of a problem is the following in this neighbourhood… |  |
| 1. Litter, broken glass, or garbage? | A big problem = 1 |
| 1. Selling or using drugs? | Somewhat a problem = 2 |
| 1. Alcoholics and excessive drinking in public? | No problem = 3 |
| 1. Groups of young people who cause trouble? |  |
