# Supplementary material for: Trajectories of Childhood Weight Gain: The Relative Importance of Local Environment versus Individual Social and Early Life Factors
Source: PLoS One. 2012 Oct 15;7(10):e47065. doi: 10.1371/journal.pone.0047065 (PMC3471956; doi:10.1371/journal.pone.0047065)
Supplement: Appendix S2 — Fit statistics for the trajectory groups estimated in the QLSCD. (DOCX) [file pone.0047065.s002.docx]

| **Appendix B: Fit statistics for the trajectory groups estimated in the QLSCD** | | | | | |
| --- | --- | --- | --- | --- | --- |
| **GROUP** | **Frequency** | **Actual Percent** | **Estimated Percent** | **Average Posterior probability** | **Odds of correct classification** |
| **1. Low-increasing** | 143 | 9.1 | 9.7 | 0.91 | 94.3 |
| **2. Low-medium, accelerating** | 580 | 37 | 36.2 | 0.86 | 10.8 |
| **3. Medium- high, increasing** | 680 | 43.4 | 43.0 | 0.88 | 9.7 |
| **4. High-stable** | 163 | 10.4 | 11.1 | 0.90 | 72.0 |
